# Human Skin Lightening Efficacy of Resveratrol and Its Analogs: From in Vitro Studies to Cosmetic Applications

**DOI:** 10.3390/antiox8090332

**Published:** 2019-08-22

**Authors:** Yong Chool Boo

**Affiliations:** 1 Department of Molecular Medicine, School of Medicine, Kyungpook National University, Daegu 41944, Korea; ycboo@knu.ac.kr; Tel.: +82-53-420-4946; 2 Brain Korea (BK) 21 Plus Kyungpook National University (KNU) Biomedical Convergence Program, Kyungpook National University, Daegu 41944, Korea; 3 Cell and Matrix Research Institute, Kyungpook National University, Daegu 41944, Korea; 4 Ruby Crown Co., Ltd., Daegu 41061, Korea

**Keywords:** resveratrol, cosmetics, skin lightening, melanin, hypopigmentation, dietary antioxidants, oxidative stress, depigmenting agent, skin aging, phytochemical

## Abstract

Antioxidants are deemed useful in controlling oxidative stress associated with extrinsic skin aging and pigmentation disorders. Resveratrol is a polyphenol compound found in many edible plants such as *Vitis vinifera*, and its inhibitory effects on the catalytic activity, gene expression, and posttranslational modifications of tyrosinase, a key enzyme in the melanin biosynthetic pathway, provide a mechanistic basis for its antimelanogenic effects seen in melanocytic cells, three-dimensionally reconstituted skin models, and in vivo animal models. As a potent antioxidant and a modulator of nuclear factor erythroid 2-related factor 2 (Nrf2), and sirtuin 1, resveratrol can also regulate multiple signaling pathways associated with inflammation and premature aging. Recent clinical studies have supported the efficacy of resveratrol and its analogs, such as resveratryl triacetate (RTA) and resveratryl triglycolate (RTG), in human skin lightening and antiaging. These findings suggest that resveratrol and its analogs are potentially useful as skin lightening and antiaging agents in cosmetics.

## 1. Introduction

Human skin is an active organ with various physiological functions [1], and its appearance contributes to personal beauty and attractiveness to other individuals [2]. Elderly people with skin that appears younger than how it should look at their actual age are reported to be more satisfied with their life [3]. The cosmetics industry is expanding because consumers are increasingly demanding highly active skin care products that can control skin aging and abnormal pigmentation [4,5]. 

Skin aging can be classified into intrinsic and extrinsic types [4,5]. Intrinsic skin aging occurs inevitably as a result of physiological changes over time, and it is highly dependent on individual genetics, ethnicity, anatomy, and hormonal status. Extrinsic skin aging is caused by various environmental factors and/or health-related factors such as ultraviolet radiation (UV) exposure, pollution, and lifestyle. Skin aging is accompanied by various invisible physiological dysfunctions and visible morphological changes [6,7]. As the skin ages, the elasticity decreases, making it appear to be sagging and wrinkled, and additionally, the skin becomes dry. In aging skin, the number of melanocytes decreases gradually, but the melanogenic activity can irregularly increase, resulting in an uneven mixture of hypopigmentation and hyperpigmentation of the skin. Both skin aging and pigmentation are important topics in dermatology and cosmetology [8,9].

Melanogenesis inhibitors are potentially useful as skin lightening agents [10]. Resveratrol is a dietary antioxidant contained in various plants such as *Vitis vinifera* L. [11,12], and evidence supporting its antimelanogenic activity has accumulated in the last decade [13,14]. As an approach to enhance the stability and efficacy of resveratrol, our research team developed its analogs, resveratryl triacetate (RTA) and resveratryl triglycolate (RTG), and undertook human trials to evaluate their skin lightening efficacy [15,16,17]. In this review, we scrutinize recent literature on the anti-melanogenic activities and skin lightening efficacies of resveratrol and its analogs to examine their potential as active ingredients for skin lightening in the cosmetics industry.

## 2. Melanin and Skin Pigmentation Disorders

Melanin pigments are classified into three basic types: (1) pheomelanin and (2) eumelanin found in the skin, hair, iris of eyes, and the stria vascularis of the inner ear, and (3) neuromelanin found in the brain. In human skin, melanin is produced in a specialized organelle called “melanosome” in the melanocytes, which localizes in the basal layer of skin epidermis [18]. Mature melanosomes filled with melanin are transferred from a single melanocyte, via dendrites, to several keratinocytes in the outer proximity, distributing melanin throughout the epidermis [19]. Melanin is an effective absorbent of UV, reducing the risk of photoaging and photocarcinogenesis [20], and is a key player in maintaining skin homeostasis [21].

The number of melanocytes per unit area of skin is similar for most people, but melanocytes of individuals from different ethnic groups produce variable amounts of pheomelanin and eumelanin, resulting in a variety of different skin color [21,22]. The distribution of melanin vertically and horizontally in the skin layers also contributes to skin color [23].

Ethnic differences in skin color are permanently determined mainly by genetic background, e.g., mutations in the SLC24A5 and SLC45A2 genes encoding the solute carrier proteins [24,25]. The single-nucleotide-polymorphisms in these genes alter the potassium-dependent sodium-calcium exchanger activity and affect melanosome biogenesis [26,27]. In addition, various non-genetic factors can affect the expression of melanin-related genes, contributing to acquired skin color changes [28]. Hormonal changes, chronic inflammation, and UV exposure are some examples of pathophysiological conditions under which disrupted melanogenesis causes hypo- or hyper-pigmentation [29].

## 3. Regulation of Melanin Synthesis

In the melanin biosynthesis, L-tyrosine or L-3,4-dihydroxyphenylalanine (DOPA) is oxidized to DOPA quinone by tyrosinase (TYR, monophenol, dihydroxyphenylalanine:oxygen oxidoreductase, EC 1.14.18.1), and these reactions are followed by multiple polymerization reactions leading to the synthesis of brownish black eumelanin or reddish-yellow pheomelanin depending on whether thiol conjugations are adopted or not [30].

Gene expression of tyrosinase and other melanogenic enzymes, such as tyrosinase-related protein 1 (TYRP1) and dopachrome tautomerase (DCT), and biogenesis of melanosomes are directed by microphthalmia-associated transcription factor (MITF) in melanocytic cells [18,31]. Proopiomelanocortin-derived peptide hormones such as α-melanocyte stimulating hormone (α-MSH), β-MSH and adrenocorticotrophic hormone regulate skin pigmentation and inflammation in response to UV and/or inflammatory stimuli [28,32]. On the binding of α-MSH to the melanocortin 1 receptor (MC1R) and subsequent activation of cyclic AMP (cAMP)-producing adenylate cyclase, cAMP-dependent protein kinase A (PKA) phosphorylates and activates cAMP-responsive element-binding protein (CREB) transcription factor, which in turn induces MITF gene expression and activation [33]. Other signaling pathways involving Wnt, glycogen synthase kinase 3β, and mitogen-activated protein (MAP) kinases can also stimulate MITF [34,35]. MITF is active in its phosphorylated form, but is degraded following ubiquitination [36]. For a more comprehensive overview of signaling pathways in melanogenesis that are not covered in this review, please refer to other recent reviews that are more focused on this topic and contain detailed schematic figures [37,38].

Unwanted abnormal skin pigmentations are clinically and aesthetically significant conditions that can cause mental stress and lower the quality of life [39]. Various approaches are used to control hyper- and hypo-pigmentation in dermatology and cosmetology. Hydroquinone is primarily used to treat hyperpigmentation in medicine, by itself or in combination with other adjuvants [40]. Various ingredients, e.g., arbutin and kojic acid are used in the cosmetics industry to control hyperpigmentation [41]. However, the satisfaction of consumers is low, and they therefore demand more effective and safer skin lightening ingredients [42,43]. A variety of natural and synthetic compounds that inhibit the catalytic activity of tyrosinase, which is a key enzyme in the melanin biosynthesis, have previously been reported in the literature [43,44], but their clinical efficacies are largely unknown.

## 4. Resveratrol: An Antioxidant with Diverse Bioactivities

Resveratrol (3,5,4′-trihydroxy-*trans*-stilbene) is a polyphenolic compound found in various plants, including grapes, berries and peanuts [45,46]. It is believed to act as a phytoalexin in several plants, providing defense from attack by insects and pathogens [47,48].

**Figure 1:**
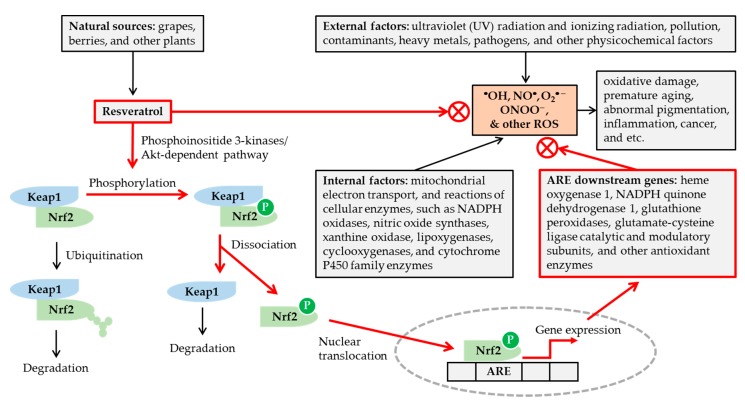
Potential mechanisms for the antioxidant action of resveratrol. Various internal and external factors can cause oxidative stress by increasing the formation of reactive oxygen species (ROS) in excess of cellular antioxidant capacity. Resveratrol, available from various natural sources, can attenuate the oxidative stress, by scavenging ROS, and/or enhancing cellular antioxidant capacity via nuclear factor erythroid 2-related factor 2 (Nrf2)-mediated mechanisms. Resveratrol stimulates phosphorylation of Nrf2 by a phosphoinositide 3-kinases/Akt (protein kinase B)-dependent mechanism, and releases Nrf2 from Kelch-like ECH-associated protein 1 (Keap1). The nuclear translocation of Nrf2 leads to the activation of antioxidant response elements (ARE) linked to gene expression of many cellular antioxidant enzymes. In this way, resveratrol can reduce oxidative damage, premature skin aging, abnormal pigmentation, and other age-related disorders.

As shown in Figure 1, excessive reactive oxygen species (ROS) formation due to internal and external factors can induce oxidative damages, inflammation, and age-related disorders. Resveratrol can act as an antioxidant and can modulate the cell functions, signal transduction and gene expression [49]. It scavenges hydroxyl radical (•OH), nitric oxide (NO•), and superoxide anion radical (O_2_•^−^), which are generated by pulse radiolysis in aqueous media and detected by transient absorption spectra of reaction intermediates [50]. It also scavenges peroxynitrite (ONOO^−^), as evidenced by the reduction in nitration of bovine serum albumin reacted with authentic peroxynitrite in solution [51].

Transcription factor, nuclear factor erythroid 2-related factor 2 (Nrf2), plays a crucial role in regulating cellular redox status [52,53,54]. Kelch-like ECH-associated protein 1 (Keap1) binds to Nrf2 under normal conditions and promotes Nrf2 degradation through the ubiquitin–proteasome pathway. Under oxidative stress, conformational changes of either Keap1 or Nrf2 lead to their dissociation. The free Nrf2 is then translocated into the nucleus and binds to the antioxidant response element (ARE) of the genes to induce the expression of many downstream genes coding for antioxidant enzymes. Resveratrol has been shown to activate the Nrf2/ARE pathway by a phosphoinositide 3-kinases/Akt (protein kinase B)-dependent mechanism [52,53,54]. It has been shown to induce nuclear accumulation of Nrf2 and gene expression of reduced nicotinamide adenine dinucleotide phosphate (NADPH) quinone dehydrogenase 1, glutathione peroxidase 2, and the catalytic and modulatory subunits of glutamate-cysteine ligase, in the primary culture of normal human keratinocytes [55].

Many biochemical properties of resveratrol are potentially useful in cosmetics as an active ingredient for preventing skin aging and pigmentation [56,57,58]. Extrinsic skin aging caused by environmental factors, such as solar radiation and pollutants, involves changes in the composition of the dermal extracellular matrix [59,60]. Matrix metalloproteinases (MMPs), which are a family of zinc endopeptidases, play a key role in the turnover of extracellular matrix macromolecules, such as type I collagen [61]. Activated MMPs in skin cells can impair the structural integrity of skin and trigger skin tissue remodeling, resulting in the formation of wrinkles and other phenotypes associated with skin aging [62]. Oral administration of resveratrol increased the protein levels of Nrf2 and heme oxygenase 1, while decreasing those of MMP1 and MMP9, in the skin of institute of cancer research (ICR) mice exposed to UV, and its efficacy was evidenced by the reduction of UV-induced skin edema and wrinkles in resveratrol treated animals [63].

Resveratrol directly or indirectly activates sirtuin 1, an NAD-dependent deacetylase, that is involved in metabolic regulation, stress response, and aging processes [64,65]. Resveratrol inhibits tumor necrosis factor (TNF)-α–induced expression of inflammatory cytokines and MMPs by a sirtuin 1-dependent mechanism [66,67]. It also attenuates the expression of inflammatory mediators induced by UV or airborne particulate matter [68,69,70]. Resveratrol can also directly inhibit the activity of MMPs [71].

## 5. Resveratrol as a Tyrosinase Inhibitor

Various stilbenoids, including resveratrol, inhibit mushroom tyrosinase activity [72,73,74]. Oxyresveratrol has been shown to exhibit more potent inhibition of L-tyrosine oxidation catalyzed by murine tyrosinase (IC_50_, 52.7 μM) than resveratrol (IC_50_ > 100 μM). Piceatannol has been shown to be a very potent inhibitor of mushroom tyrosinase (IC_50_, 1.53 μM), compared to kojic acid (IC_50_, 50.1 μM) and resveratrol (IC_50_, 63.2 μM) [75]. Oxyresveratrol is found in many plants, such as *Morus alba*, and shows antioxidant activity mitigating oxidative stress and inflammatory reactions [76,77]. Anthraquinones from *Polygonum cuspidatum*, such as physcion, emodin, citreorosein and anthraglycoside B) have also been shown to be more potent inhibitors of mushroom tyrosinase than resveratrol and piceid (resveratrol 3-β-mono-D-glucoside) [78]. Gnetin C, a resveratrol dimer isolated from melinjo (*Gnetum gnemon*) has been shown to be as effective as resveratrol with regard to its inhibitory activity against mushroom tyrosinase, but the former has a much weaker inhibitory activity against murine tyrosinase than the latter [79]. *Vitis vinifera* extracts containing gallic acid, chlorogenic acid, epicatechin, rutin, and resveratrol show competitive inhibition against mushroom tyrosinase activity [80]. Collectively, these studies suggest that resveratrol is a modest, and not very potent, inhibitor of mushroom tyrosinase.

Although mushroom tyrosinase, which is commercially available in a purified form, has been widely used as a substitute for human tyrosinase, it is significantly different from human tyrosinase in terms of amino acid sequence [81,82]. Tyrosinases derived from different organisms have been shown to be inhibited to markedly different extents by a single inhibitor [83,84]. Thus, it is unreasonable to expect that these mushroom tyrosinase inhibitors would be effective for human skin lightening through the same action without direct evidence.

Our team has developed a cell line transformed from human embryonic kidney (HEK) 293 cells to constitutively express human tyrosinase [85,86]. Using the cells as a source of human tyrosinase, various compounds and plant extracts were tested for their inhibitory effects on human tyrosinase activity [85,86,87,88]. As a result, several compounds, such as *p*-coumaric acid, were found to strongly inhibit human tyrosinase [85,89]. Resveratrol has been shown to be an active component of *Vitis vinifera* extracts that inhibit human tyrosinase activity [87]. Resveratrol inhibited human tyrosinase activity more strongly (IC_50_, 0.39 µg mL^−1^) than p-coumaric acid (IC_50_, 0.66 µg mL^−1^) and arbutin (IC_50_ > 100 µg mL^−1^). Resveratrol had much lower effect on mushroom tyrosinase activity than on human tyrosinase activity. 

Resveratrol can be biotransformed by mushroom tyrosinase to its oxidized form, which is a more powerful inhibitor of mushroom tyrosinase than resveratrol itself [90,91,92]. The reaction products of resveratrol by tyrosinase were more toxic than resveratrol itself [93]. Oxyresveratrol is also a substrate of mushroom tyrosinase [94]. Thus, it is necessary to study whether the same mechanism also applies to human tyrosinase. The potential cytotoxic effects of resveratrol and its metabolites would be an important topic for future studies, keeping in mind the recent social impacts observed in the case of rhododendrol, another depigmenting agent [95].

## 6. Other Antimelanogenic Mechanisms of Resveratrol and Its Analogs

Although resveratrol inhibited tyrosinase activity less effectively than oxyresveratrol in vitro, the former inhibited cellular melanogenesis more effectively than the latter [96]. When resveratrol was used in combination with 4-n-butyl resorcinol or oxyresveratrol, they synergistically inhibited tyrosinase activity and tyrosinase gene expression [97,98]. Various chemical modifications have been attempted to enhance the therapeutic potential of resveratrol [99,100]. Some chemically synthesized resveratrol analogs showed more potent inhibition of tyrosinase activity, tyrosinase gene expression, and/or cellular melanin synthesis than resveratrol demonstrated [101,102,103,104]. Semi-synthetic derivatives from resveratrol showed altered inhibition against tyrosinase activity and cellular melanin synthesis [96,105,106,107].

MITF binds to the E-box (CAYRTG) and M-box (TCAYRTG or CAYRTGA) sequences in the promoter regions of target genes such as MC1R, tyrosinase, TYRP1 and DCT, and transactivates these genes [108]. Resveratrol inhibited MITF promoter activity induced by UV or forskolin in B16 cells [109]. Resveratrol, resveratryl triacetate (RTA), and resveratryl triglycolate (RTG) lowered the mRNA and protein levels of tyrosinase, DCT and MITF in human epidermal melanocytes [96,106]. Resveratrol and its trimethyl ether decreased the tyrosinase protein level and tyrosinase activity in B16 cells stimulated by α-MSH [107]. Therefore, resveratrol and its analogs are assumed to reduce the gene expression of MITF and downstream melanogenic enzymes by inhibiting the cAMP-dependent pathway.

Resveratrol activates sirtuin 1, which in turn activates transcription factors p53 and forkhead box O (FOXO) [110]. Resveratrol has been shown to increase both sirtuin 1 and FOXO3a in human melanocytes [111]. The inhibitory effects of resveratrol on the expression of MITF and tyrosinase were not affected by sirtuin 1 inhibitor but was reduced by c-Jun N-terminal kinase (JNK) inhibitor that also modulates FOXO3a. Thus, although additional direct evidence is needed, it has been suggested that resveratrol can confer the antimelanogenic activity through a FOXO3a-dependent mechanism [111]. Resveratrol is also known as a potent inducer of autophagy [112], which is a lysosome-dependent mechanism for removing misfolded or damaged proteins or unnecessary organelles [113]. Resveratrol increased expression levels autophagy-related gene 5 (ATG5) while decreasing MITF, tyrosinase, and TYRP1 in Melan-A cells stimulated by α-MSH [114]. Small interfering RNA-mediated depletion of ATG5 rescued the expression of MITF, tyrosinase, and TYRP1 in the presence of resveratrol, indicating that autophagy is associated with the antimelanogenic effects of resveratrol.

**Figure 2:**
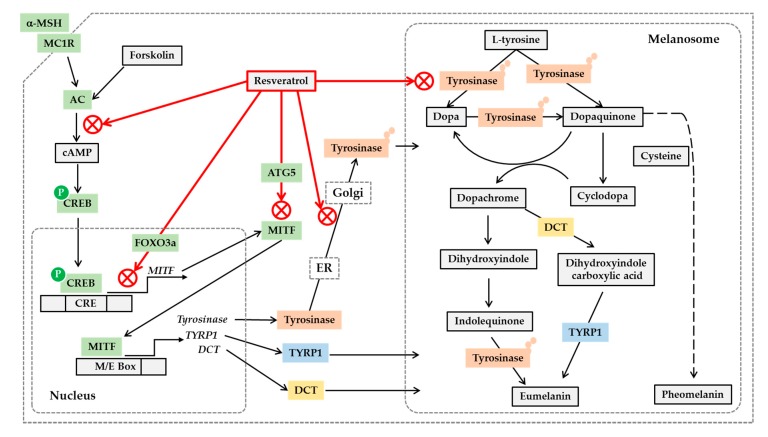
Potential mechanisms for the antimelanogenic action of resveratrol. On the binding of α-melanocyte stimulating hormone (α-MSH) to the melanocortin 1 receptor (MC1R) and subsequent activation of cyclic AMP (cAMP)-producing adenylate cyclase (AC), leads to the phosphorylation of cAMP-responsive element-binding protein (CREB) by protein kinase A. Phosphorylated CREB enters nucleus and binds to cAMP response elements (CRE) on the promoter of its target genes including microphthalmia-associated transcription factor (MITF), activating their gene expression. Resveratrol can inhibit the gene expression of MITF, tyrosinase, tyrosinase-related protein 1 (TYRP1) and dopachrome tautomerase (DCT) stimulated by α-MSH or forskolin, a director activator of AC, which is mediated by a cAMP-dependent mechanism. Resveratrol can suppress MITF activation by a FOXO3a-dependent mechanism. Resveratrol can also stimulate autophagy-related gene 5 (ATG5) expression inducing autophagy, and reduce the protein levels of MITF and tyrosinase. The antimelanogenic enzymes such as tyrosinase undergo posttranslational modifications in the endoplasmic reticulum (ER) and Golgi, and resveratrol can inhibit these processes. Resveratrol can also inhibit enzyme reactions of tyrosinase involved in the synthesis of eumelanin and pheomelanin in the melanosomes. Although the synthetic route of pheomelanin is simply drawn here, it is very complicated and involves many enzymes and metabolites. There are many other pathways that are involved in the regulation of cellular melanin synthesis, but are not covered in this figure.

Post-translational modifications of tyrosinase and other melanogenic enzymes are required for full activation [115,116]. Normal human melanocytes contain mainly the mature, Golgi-processed form of tyrosinase, but the cells treated with resveratrol contain mostly endoplasmic reticulum (ER)-retained, immature tyrosinase. This indicates that resveratrol can disrupt trafficking of tyrosinase from the ER to the Golgi and maturation of tyrosinase [117]. Thus, resveratrol and its analogs are considered to regulate cellular melanin synthesis by multiple mechanisms, including the inhibition of catalytic activity, gene expression, and posttranslational maturation of tyrosinase in melanocytes. The potential anti-melanogenic action mechanism of resveratrol is shown in Figure 2.

Resveratrol has been reported to rather increase melanin synthesis in melanoma cells [118]. In this study, resveratrol inhibited cell proliferation and increased melanin synthesis, which was accompanied by increased tyrosinase activity. Phosphorylation of CREB increased and phosphorylation of extracellular signal regulated kinase (ERK) decreased, but MITF remained unchanged. Because tyrosine protein levers were not shown in this study, the precise mechanism for this phenomenon is currently uncertain.

## 7. Hypopigmentation Effect of Resveratrol

In vivo experiments and human tests on the skin lightening and antiaging activity of resveratrol and its analogs are listed in Table 1. In dark-skinned Yucatan swine, topical treatment with 1% resveratrol twice a day, 5 days per week, for 8 weeks resulted in visible skin lightening without signs of irritation or other undesired effects [109]. In another experiment using light-skinned Yucatan swine, skin tanning was induced by exposing them to one minimal erythema dose (MED) of UVB, once per day, on three alternate days. Topical treatment with 1% resveratrol once daily for 2 weeks, immediately after each UVB exposure and on non-UVB exposure days, reduced the UVB-induced pigment deposition in Yucatan swine.

Lee et al. have tested the hypopigmentation effect of resveratrol in brownish guinea pigs [13,119,120]. In one study [13], pigmentation was induced by exposing the dorsal skin of guinea pigs to UVB (λ_max_, 310 nm) at 390 mJ cm^−2^ thrice per week, for two weeks, and thereafter, 1% resveratrol solution was topically applied every day to these animals for 2 weeks. As a result, UVB exposure increased the pigment index from 40.7 ± 1.6 in the base-line group to 62.6 ± 2.3 in the vehicle control group and 53.4 ± 1.0 in the 1% resveratrol treatment group, indicating a hypopigmentation effect of resveratrol. Histological data suggested that resveratrol reduced melanin synthesis by decreasing DCT among the melanogenic enzymes. In subsequent studies, resveratrol-enriched rice extract and the same extract encapsulated in nanoparticles were shown to exhibit hypopigmentation effects in guinea pigs [119,120].

## 8. Human Skin Lightening Efficacy of Resveratrol

The effects of resveratrol against skin pigmentation and sunburn caused by repetitive UV irradiation were examined in a human trial employing 15 healthy volunteers [14]. Six sites on the non-exposed dorsal skin of each volunteer were exposed to solar simulating UV at a dosage of 1.5 MED for 4 consecutive days, and different test materials were topically applied immediately after each UV exposure.

The skin color can be expressed using the Commission Internationale de l’Eclairage Lab color space composed of the degree of lightness (L*), degree of green to red (a*), and degree of yellow to blue (b*) [122]. In this study, the skin color parameters, L*, a*, and b were measured using Spectrophotometer^®^ CM-2500d (Minolta, Tokyo, Japan) [122].

Four days after UV irradiation, L* values decreased from 63.89 to 55.91 in the control group, and from 64.20 to 59.3 in the 1% resveratrol treatment group, indicating reduced tanning in the treatment group. The a* values increased from 7.62 to 16.29 in the control group, and from 7.51 to 13.43 in the treatment group, indicating that sunburn was reduced in the treatment group. Histological analysis supported that UV-induced sun burn and sun tan were reduced by resveratrol treatment.

## 9. Human Skin Lightening Efficacy of Resveratryl Triacetate (RTA)

In cosmetics, not only the efficacy and safety of the active ingredient, but also its stability, are important considerations; however, resveratrol is not stable enough to be used in cosmetics [123,124]. Various approaches have been developed to enhance its stability in cosmetic formulations [123,125]. As an approach to improve the stability of resveratrol as an active ingredient in cosmetics, resveratrol was acetylated to RTA as a “prodrug” form [96,126]. RTA showed higher stability in solutions, lower cytotoxicity, and similar inhibitory effect on melanin synthesis in cultured melanocytes, as compared to resveratrol itself [96]. It is assumed that the acetylated compound may be converted to resveratrol by the esterase enzymes in cells.

The safety and skin lightening efficacy of RTA were investigated in human studies [15,16]. The primary skin irritation potentials of resveratrol and RTA were assessed at 0.1% and 0.5% concentrations in thirty three healthy women [15], via a closed patch testing method [127,128]. Inert Quadrate (IQ) chambers loaded with a test material were applied to the test sites on the dorsal skin of each volunteer for 48 h, and the occurrence of adverse skin reactions was examined at 30 min and 24 h after the patch was removed from the skin; the overall irritation potentials were graded using the criteria described by Frosch and Kligman, with a slight modification [129,130]. On testing, resveratrol was observed to induce weak skin irritation at 0.5%, whereas RTA did not induce any adverse skin reactions [15].

The human skin lightening efficacy of RTA was evaluated [15] using the artificial tanning and natural hyperpigmentation models [131,132]. The color parameters, L*, a*, and b were measured using Spectrophotometer^®^ CM-2500d (Minolta) [122,133]. The skin color was represented by the individual typology angles (ITA°) which were calculated using the equation: ITA° = (arc tangent [(L* − 50)/b*]) 180/3.14159 [134]. The higher the ITA° value, the lighter the skin color.

In all, 22 women with Fitzpatrick skin types III or IV were enrolled in the test using the artificial tanning model. Skin tanning was induced by exposing two test sites (15 mm × 15 mm) on the forearms of each volunteer to UV from a solar simulator at 2 MED and waiting for 7 days. The volunteers were randomly divided into two groups, and depending on the group, each of the two test sites in each volunteer received either the test product containing 0.4% RTA or the control product, twice daily, for 8 weeks.

As tanned skin underwent the depigmentation process, the ITA° increased continuously for 8 weeks, in both test and control groups. The application of the test and control products for 8 weeks increased the ITA° by 17.60% and 13.81%, respectively, and the difference was statistically significant (*p* < 0.05). In another study using the natural hyperpigmentation model, 21 women were enrolled. The volunteers were divided into two groups and depending on the group, the right or left sides of the face of each volunteer received either the test product containing 0.4% RTA or the control product, twice daily, for 8 weeks. The pigmentation intensity of the highly pigmented area decreased by 2.67% and 1.46% in the test and control groups, respectively, and the difference was statistically significant (*p* < 0.05). These studies supported the human skin lightening efficacy of topically applied 0.4 % RTA.

The human skin lighting efficacy of 0.8% RTA was further examined using the artificial UV-induced tanning model in a separate study [16]. In this study, 23 women volunteers were enrolled, and the artificially tanned forearm skins of each volunteer received either the test product containing 0.8% RTA or the control product, twice daily, for 8 weeks. As an index of skin color, ITA° increased continuously for 8 weeks in both the test and control groups. The test product containing 0.8% RTA and control products increased ITA° by 20.38% and 16.31% after 8 weeks, respectively, indicating that depigmentation was faster in the test group than in control group. Visual assessment of the pigmentation degree was conducted by two experienced examiners, using a pigmentation index from 0 (bright and transparent) to 9 (dark and dull), in increments of 0.5. The pigmentation degree had decreased continuously for 8 weeks in both test and control groups. The pigmentation degrees decreased in the test and the control group by 35.79% (from 7.07 to 4.54) and 30.93% (from 7.08 to 4.89) after 8 weeks, respectively. The intergroup difference was statistically significant (*p* < 0.05). Therefore, 0.8% RTA-containing cosmetic products can confer skin lightening efficacy in humans.

## 10. Human Skin Antiaging Efficacy of Resveratryl Triacetate (RTA)

The human skin antiaging efficacy of 0.8% RTA-containing cream was examined in a study involving instrumental analyses of facial skin wrinkles, sagging, elasticity, dermis denseness, moisture, and brightness [121]. In all, 20 women volunteers were enrolled in the study and they used the test product containing 0.8% RTA on their face twice daily (morning and evening) for 8 weeks. 

The skin wrinkles around crow’s feet were evaluated using a three-dimensional image analyzing system (PRIMOS^®^ Premium, GFMesstechnik GmbH, Teltow, Germany) [135]. The sagging of cheek was evaluate by analyzing a Moire pattern image, taken by F-ray^®^ (Beyoung, Seoul Korea), using Image-pro^®^ plus (MediaCybernetics, Rockville, MD, USA) [136]. The elasticity of cheek skin was evaluated by a suction method using Cutometer^®^ MPA580 (Courage + Khazaka electronic GmbH, Cologne, Germany) [137]. The denseness of cheek dermis was evaluated using ultrasound images generated by DermaLab^®^ Series SkinLab Combo (Cortex Technology, Hadsund, Denmark) [138,139]. The moisture content of cheek skin was measured using Corneometer^®^ CM 825 (Courage + Khazaka electronic GmbH) [140].

Compared with the baseline values before treatments, total wrinkled area decreased (5.12%, 4.86%), total wrinkle volume decreased (10.53%, 8.41%), sagging decreased (4.69%, 5.91%), elasticity increased (2.84%, 3.98%), denseness increased (15.65%, 20.80%), moisture content increased (5.83%, 7.37%), lightness (L* value) increased (0.79%, 1.07%), and ITA° (a skin color index) increased (5.43%, 4.95%) after 4 and 8 weeks of using the test product. Changes of all these parameters were statistically significant (*p* < 0.05), and no adverse skin reactions were observed in any participant during the entire study period. This study supports the skin anti-aging efficacy of RTA, although other ingredients contained in the test products may also have contributed to the efficacy.

## 11. Human Skin Lightening Efficacy of Resveratryl Triglycolate (RTG)

RTG is a new hybrid compound between resveratrol and glycolic acid [106]. RTG is different from RTA, in that resveratrol is chemically coupled to glycolic acid in RTG instead of being coupled to acetic acid in RTA. Glycolic acid is a kind of α-hydroxy acid widely used in skin care products for various purposes including chemical peeling of dull skin [141,142]. Compared with RTA that is very hydrophobic, RTG is moderately soluble in water because of the polar hydroxyl group. The resveratryl moiety of RTG was expected to reduce the production of new melanin and the glycolic moiety was expected to remove the keratin that previously accumulated melanin. RTG inhibited tyrosinase activity in vitro and MITF and tyrosine gene expression, suppressing cellular melanin synthesis as effectively as, or slightly more effectively than, resveratrol and RTA [106].

Primary skin irritation potential of RTG was tested in a human study where 30 healthy women participated [17]. The test product contained 0.4% RTG and the control product comprised the same formula without RTG. In patch testing, neither of the control product nor the test product containing 0.4% RTG induced any adverse skin reactions in any participant.

The depigmenting efficacy of RTG was tested in a human trial using a UV-induced artificial tanning model [17]. In this trial, 22 women volunteers with Fitzpatrick skin type III or IV were included. Tanning was induced by UV exposure of the designated skin sites in the forearms of each volunteer and waiting for 7 days, and thereafter the test product containing 0.4% RTG or the control product was applied at 20 μL cm^−2^ twice daily for 8 weeks. Melanin index, the absorptivity of the pigments at specific wavelengths, was measured using Mexameter^®^ MX18 (Courage + Khazaka electronic GmbH), in which the probe emits 3 specific light wavelengths (green: λ = 568 nm, red: λ = 660 nm, and infrared: λ = 880 nm), and a receiver measures the light reflected by the skin.

The melanin index decreased from 205 to 163 in the test group and to 172 in the control group, and the difference between these two groups was statistically significant (*p* < 0.05). Analysis of ITA° as a skin color index showed that the test product containing 0.4% RTG and the control product increased ITA° by 24.42% and 17.81% in 6 weeks and by 28.96% and 22.06% in 8 weeks, respectively. The intergroup differences at each time point were statistically significant (*p* < 0.05). The pigmentation degrees visually assessed by two experienced examiners were also supportive for the clinical efficacy of RTG. The test product containing 0.4% RTG and the control product decreased the pigmentation degree by 31.9% (from 7.05 to 4.98) and 29.4% (from 7.11 to 4.84) in 8 weeks, respectively. The intergroup difference was statistically significant (*p* < 0.05).

## 12. Conclusions and Perspectives

In conclusion, the results from in vivo studies or clinical studies are supportive of the human skin lightening and/or antiaging efficacies of resveratrol and its analogs. In animal studies and in clinical trials, 1% resveratrol has been shown to reduce pigmentation induced by UV when it is applied topically to the skin. Resveratrol is considered to attenuate cellular melanin synthesis through inhibition of tyrosinase catalytic activity, and inhibition of processes such as tyrosinase gene expression, tyrosinase protein maturation, and autophagy. Although there is a lack of direct evidence, resveratrol might interfere with melanosome biogenesis because it reduced the activity of MITF, which is a key regulator of melanosome biogenesis as well as melanogenesis.

The resveratrol analogs, RTA and RTG, also showed human skin lightening effects in clinical trials at the tested concentrations (04% RTA, 0.8% RTA and 0.4 % RTG). Moreover 0.8 % RTA showed antiaging effects improving various skin parameters such as facial skin wrinkles, sagging, elasticity, dermis denseness, moisture, and brightness. Comparison of separate clinical studies with regard to the skin lightening efficacies of RTA and RTG indirectly suggests that the efficacy of RTG is slightly superior to that of RTA. RTA and RTG might act as “prodrugs” of resveratrol, and their skin lightening efficacies would depend on their penetration through the skin and biotransformation to resveratrol in cells.

**Figure 3:**
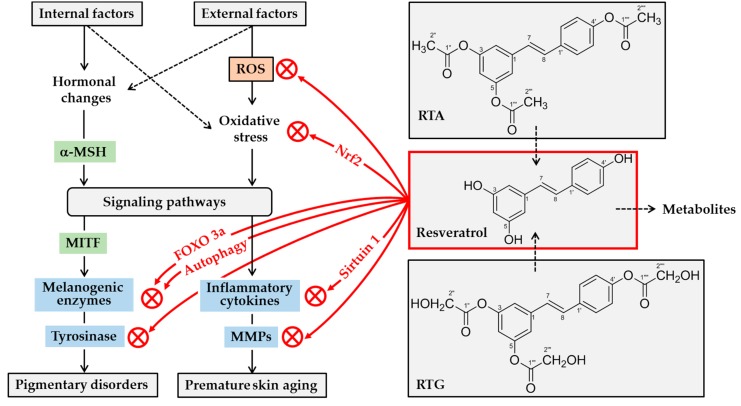
Potential mechanisms for skin lightening and antiaging actions of resveratrol. A variety internal and external factors can induce hormonal changes and oxidative stress, stimulating multiple signaling pathways linked to gene expression of melanogenic enzymes and inflammatory mediators. α-Melanocyte stimulating hormone (α-MSH) stimulates gene expression of melanogenic enzymes by microphthalmia-associated transcription factor (MITF)-dependent mechanism. Resveratrol can attenuate oxidative stress of cells either by decreasing levels of reactive oxygen species (ROS), or by increasing antioxidant capacity via nuclear factor erythroid 2-related factor 2 (Nrf2). It can suppress the expression of inflammatory cytokines and matrix metalloproteinases (MMPs) by directly or indirectly activating sirtuin 1, an NAD-dependent deacetylase. It can directly inhibit the catalytic activity of MMPs. It can inhibit tyrosinase gene expression by multiple mechanisms involving either the activation of forkhead box O 3a (FOXO3a) or autophagy. Further, it can inhibit tyrosinase protein maturation, and/or tyrosinase catalytic activity. Topical application of resveratrol or its analogs, such as resveratryl triacetate (RTA) and resveratryl triglycolate (RTG), can reduce pigmentation and/or skin aging processes. Metabolites of resveratrol may have negative or positive effects on skin health before they are excreted from the body.

Based on the current understanding, resveratrol can whiten human skin and retard skin aging by a number of mechanisms: (1) direct inhibition of the catalytic activity of human tyrosinase, (2) suppression of gene expression and maturation of tyrosinase and other melanogenic enzymes, (3) direct scavenging of ROS and/or inhibition of their production, (4) enhancement of cellular antioxidant capacity through Nrf2-dependent mechanisms, (5) attenuation of inflammatory responses of cells, and (6) direct inhibition of the catalytic activity of MMPs. This concept is graphically depicted in Figure 3.

Future studies will need to technically improve the clinical efficacy of resveratrol and its analogs to be more satisfactory to the consumers of cosmetics, notably by increasing their content in cosmetic products and by enhancing their skin permeation via optimized formulations. Higher efficacy may be expected from a combinatory use of them with other active ingredients modulating different targets in the skin pigmentation process, such as intercellular melanosome transfer from melanocytes to keratinocytes [143,144,145]. Future studies are also needed to critically examine the safety of resveratrol, its analogs, and their metabolites.

## Figures and Tables

**Table 1:** In vivo and clinical studies on the skin lightening efficacy of resveratrol and its analogs.

Literature	Tests	Models	Treatments	Assessments
Lin et al., 2002 [109]	Yucatan swine	Natural pigmentation	1% Resveratrol	Visual Evaluation
		UV-induced tanning		
Lee et al., 2014 [13]	Guinea pigs	UV-induced tanning	1% Resveratrol	Instrumental methods
				Visual Evaluation
Wu et al., 2013 [14]	Humans	UV-induced tanning	1% Resveratrol	Instrumental methods
Ryu et al., 2015 [15]	Humans	UV-induced tanning	0.4% RTA	Instrumental methods
		Natural pigmentation		
Boo, 2016 [16]	Humans	UV-induced tanning	0.8% RTA	Instrumental methods
				Visual Evaluation
Ryu et al., 2018 [121]	Humans	Natural pigmentation	0.8% RTA	Instrumental methods
Ryu et al., 2018 [17]	Humans	UV-induced tanning	0.4% RTG	Instrumental methods
				Visual Evaluation

Abbreviations: RTA, resveratryl triacetate; RTG, resveratryl triglycolate.
